# Patients' roles in governance of learning: Results from a qualitative study of 16 learning healthcare systems

**DOI:** 10.1002/lrh2.10269

**Published:** 2021-05-25

**Authors:** Rachel Grob, Katharine Gleason, Paul McLean, Sarah McGraw, Mildred Solomon, Steven Joffe

**Affiliations:** ^1^ Center for Patient Partnerships University of Wisconsin‐Madison Madison Wisconsin USA; ^2^ Department of Medical Ethics and Health Policy University of Pennsylvania Philadelphia Pennsylvania USA; ^3^ Independent patient/family co‐investigator; ^4^ The Hastings Center Garrison New York USA; ^5^ University of Pennsylvania, Philadelphia Pennsylvania USA

**Keywords:** governance, learning health systems, patient engagement

## Abstract

Patient and family engagement has been identified as key to fulfilling Learning Healthcare Systems' (LHSs') promise as a model for improving clinical care, catalyzing research, and controlling costs. Little is known, however, about the state of patient engagement in the learning mission of these systems or about what governance structures and processes facilitate such engagement. Here, we report on an interview study of 99 patient and employee leaders in 16 systems. We found both variable levels of engagement and broad agreement that shared governance of learning remains a work in progress. We also identified a range of practices that can support or thwart development of an organizational culture conducive to shared governance, including transparency, capacity building, infrastructure investment, leadership, attention to diversity of patient partners, and committee structures. In LHSs with most sophisticated shared governance, both employees and patients contribute to building a democratic learning culture.

## 1 INTRODUCTION

For more than a decade, the National Academy of Medicine (NAM, formerly Institute of Medicine), the Agency for Health Research and Quality (AHRQ), and numerous scholars have called on health systems to become Learning Healthcare Systems (LHSs).^1^, ^2^, ^3^, ^4^, ^5^, ^6^ LHSs are defined as systems “in which knowledge generation is so embedded into the core of the practice of medicine that it is a natural outgrowth and product of healthcare delivery and leads to continual improvement in care.”^1^ They strive to develop evidence‐based practices and to create a culture of learning. They iteratively study how they care for patients in real time, use what they learn to improve care, implement proven practices system wide, and disseminate findings.^1^, ^7^, ^8^ Some evidence suggests that systems further along in this evolution do indeed show improvements in quality and cost.^9^

Central to conceptions of a robust LHS is commitment to patient participation in governance of learning, which enables patients to contribute as partners throughout the learning cycle. ^1^, ^2^ AHRQ, for example, emphasizes that LHSs must “promote the inclusion of patients as vital members of the learning team.”^2^ Scholars also consider engagement essential to demonstrating respect for patients in the LHS environment where, for many learning activities, the roles of institutional human subjects research review and informed consent are diminished compared with traditional research. ^3^, ^10^ In other words, LHSs cannot fully accomplish their missions without a strategy to robustly engage patients and families.

Despite growing investment in LHSs, little empirical research addresses the actual status of patient engagement within them or what leadership and organizational features facilitate it.^11^ In particular, little is known about the roles that patients play, not just in individual learning activities, but also in governance of the learning enterprise as a whole. By governance, we mean the set of structures and processes that ensure that activities are consistent with the organization's learning mission and related values. These might include—but are not limited to—prioritization, selection of learning approaches, protection of patients and research participants, assurance of methodological quality, and internal implementation and external dissemination of findings.^12^ We consider patient engagement in governance to be substantive if it is sufficiently robust to influence which learning activities are done, how they are conducted, or how their results are implemented or disseminated.^13^

Some relevant insights about patient involvement in learning emerge from research on discrete LHS functions such as quality improvement (QI) and research. The QI literature documents critical challenges to meaningful involvement of patients including recruitment and retention, time commitment, lack of shared knowledge and experience, socio‐cultural barriers to partnership, and the difficulty of implementing change within complex systems.^14^, ^15^ The literature on engaging patients in research is growing, ^16^, ^17^, ^18^ but with few exceptions, strategies and typologies have not been tested or adapted for engagement specific to the governance functions within LHSs. In one notable single‐institution study, Kraft et al document how patient engagement can be “hard‐wired” by, for example, establishing Patient/Family Advisory Councils (PFACs), training, and protocols for volunteers.^19^ Another case study documents how engaging patients and community members in a research network presents challenges and opportunities distinct from those associated with engagement in discrete learning activities.^20^ Such exceptions notwithstanding, the LHS literature emphasizes the need for patient engagement but provides little insight about how to achieve this goal or what practices make shared governance with patients possible.

This paper draws on qualitative interviews with 99 employee and patient/family leaders in diverse positions at 16 LHSs around the United States to explore the critical nexus^5^ where patient engagement, LHSs, and governance practices meet. It adds to the existing literature on patient engagement with QI and research by looking specifically at how patients and families are sometimes engaged in but more often excluded from governance of structured learning activities and continuous improvement processes undertaken by the LHSs we studied. It also elucidates structural arrangements within LHSs that facilitate or impede meaningful engagement at the level of organizational design and governance.^21^ Resulting insights can assist patient leaders, health systems, and policymakers as they strive to realize LHS aspirations.

## 2 METHODS

### 2.1 Overview

We conducted a qualitative analysis of how patients and family members are engaged in the governance of systematic learning at 16 purposively sampled LHSs based on telephone interviews with 20 patient/family leaders and 79 employee leaders.

### 2.2 LHS and participant selection

The 16 LHSs were identified as learning systems by key informants, their websites, or peer institutions. To ensure geographic and institutional diversity, we sought to include LHSs from four geographic regions of the United States (West, Central, South, and Northeast), academically affiliated and nonaffiliated institutions, children's hospitals, and safety‐net institutions (see Exhibit 1).

**EXHIBIT 1:**
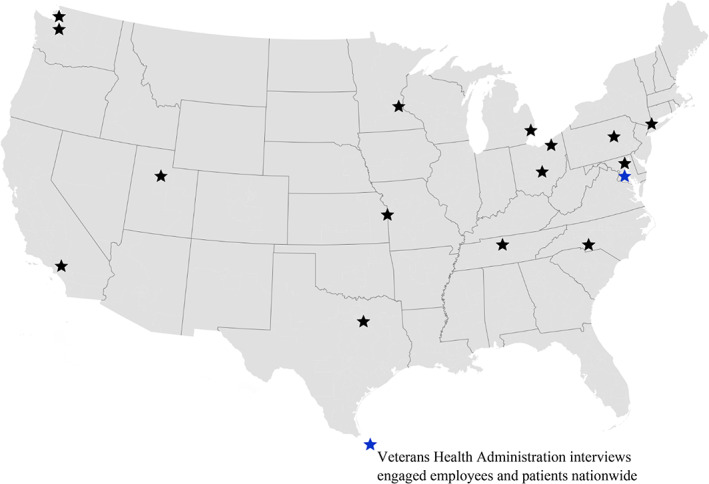
Location of learning health systems included in study

We identified employee leaders through the LHS website and internal referral or snowball sampling. Employee leaders, in turn, referred us to patients and advisors whom they considered most central to their organization's governance of learning. Most patients were members of Patient and Family Advisory Committees (PFACs), whereas some were associated with external advocacy groups or served on standing committees alongside staff. Employee leaders were members of the administration with responsibility for the governance of learning activities. Exhibit 2 provides additional detail regarding roles of study participants.

**EXHIBIT 2:** Interview participants by role category

Role	Total
Patient/Family Leader	20
Director of Quality Improvement	13
Chief Medical Officer	11
Head of Research Institute	10
IRB Director	8
Chief Experience Officer	8
Chief Information Officer	8
Chief Nursing Officer	7
Chief Executive Officer	6
Other^a^	8
	99

^a^ includes human resources personnel, system educators, and other similar position.

### 2.3 Data collection

We developed two interview guides, one for employee leaders and one for patient/family leaders. Source material for developing the guides included review of the literature on governance, patient engagement, and components of learning as articulated in the learning health care systems and patient engagement literatures.^11^, ^12^, ^19^, ^22^ Because it was relevant to our goal of understanding levels of patient engagement in governance of learning, we designed specific probes around elements of the learning cycle in learning health care systems, that is, setting priorities, designing and conducting learning, spreading successful learning across the system, and disseminating findings to other systems. Because the existing literature highlights ethical concerns with respect to the boundary between quality improvement and research in LHSs, we also included probes about strategies for protecting the rights and interests of patients.

We conducted interviews between July 2017 and November 2018. Given the differences in perspective and experiences between employees and patient advisors, we created separate but overlapping interview guides. Participants gave verbal consent for the interview and were offered a $75 gift at interview completion. The University of Pennsylvania institutional review board (IRB) approved the study.

### 2.4 Qualitative analysis

Thematic analysis proceeded in stages. Initial coding of the 99 interviews combined inductive (emergent codes) and deductive (codes defined a priori by the study questions) approaches.^11^, ^22^, ^24^ Axial coding related specifically to patients' roles in governance and then focused on a subset of parent codes: (a) the structure, process, and extent of patient/family involvement in LHS governance and (b) the impact and influence of patients/families within the LHS. Three authors (RG, KG, and PM) then discussed the themes emerging from these parent codes, developed and applied a new set of axial codes, and analyzed the resulting reports to identify patterns of patient engagement related to governance of learning. For our sub‐analysis of the range of governance functions in which patients participate (first section of findings, below), we drew on axial codes focused on the roles patients and families play, and the substance with which they engage, thus deriving a specific view of their participation in various components of learning. The conceptual models we developed as analysis proceeded elaborate Carman et al's multidimensional framework for patient and family engagement in health and health care^21^; through an iterative process of comparing inductively derived insights from our own interview data against that framework, we created a new model summarizing levels of patient engagement in governance of learning (see Exhibit 4).

### 2.5 Stakeholder engagement

The research team included a patient member from project inception through all stages of implementation and dissemination. A Stakeholder Advisory Board composed of five patient leaders and five academic experts met quarterly to advise the research team.

### 2.6 Limitations

This analysis does not aim to compare the 16 LHSs from which interviewees were drawn. Although we were sometimes able to describe a system's processes or approach by triangulating data from multiple interviewees within one LHS, we generally used individual respondents as the unit of analysis. A limited number of individuals were interviewed at each LHS (median 7, range 1‐10). Our findings thus represent the views of select individuals at 16 specific healthcare systems at a specific point in time and may not comprehensively reflect broader perspectives and experiences. Because we did not independently analyze metrics or outcome measures from participating LHSs, assessments of what strategies are and are not effective are based solely on individual interview data. Finally, while this study illuminates processes related to development of collective patient voice, it offers only limited data focused on diversity of patients' voices and barriers to it.

## 3 RESULTS

### 3.1 Patients participate in a range of governance functions within LHSs

Interviewees described numerous examples of patient involvement in governance across the life cycle of learning; Exhibit 3 provides illustrative interview excerpts for each phase.

**EXHIBIT 3:** Patient engagement in governance through the learning cycle

Setting priorities	**Patient** But um, you know we did a little bit of research and how [another LHS] got to 100 percent for hand washing, and we [patients], as a group, wrote a letter to nursing leadership and the president of the hospital and we got very vocal about it, and we have had 7 months now where we have 99 percent hand washing, and we feel like we've really influenced that. We worked with readmissions teams and we actually rewrote the criteria for the expectation of the nurse who was doing the readmissions. We looked at their satisfaction results. **Employee** We've developed a method of engagement called the community engagement studio, and it's a model where we get eight to 12 people from the condition or community impacted to come to the table to give us input on the project. It's different from a focus group in that the intent is not qualitative research, but the intent is to inform the study design.
Designing and conducting learning activities	**Patient** Okay, so for example, they want safety… they're all about, “Okay, how can we reduce fall risks? What are measures that we can take?” [So] we are going to work with our staff and brainstorm—for lack of better terminology—about how that's going to happen and then we're gonna get advice from council people like me… to see if that could be implemented and if it's practical being implemented at the hospital or in the clinics and then, if it's so, then are we gonna do measurable outcomes from it…” **Employee** So if a study's gonna happen in a certain community, patients are involved in a committee to develop the data collection instruments, the recruitment approach, and things of that sort.
Protecting patients' rights and interests	**Patient** There's less education for community stakeholders. There're probably only a handful of us that really understand what the learning healthcare system is in [city]. And then, of course, there're all of the communities that never walk through the [organization] system. And, so, we're always trying to figure out more ways to get out more information so that there's proactive informed consent, and, so, that it inspires and provides access to and mobilizes communities to learn more about this so they can be assured that if they're in a pragmatic study, that there's certainly no to low risk but lots of potential benefit. Versus traditional research, where they would be normally—they'd be consented before they ever participated in a trial. **Employee** And then the only thing more that [the Community Advisory Board] talked about was how could we broadly inform the patients that come to [organization] about these [learning activity type] programs? They accepted that informed consent was not practicable, and they did not think even necessary, but they did think some level of transparency with the patient population that these studies were going on would be needed, and so we talked about various ways of doing that and putting brochures in waiting room areas where studies are typically done. In some studies, we've actually posted a notice on the wall as you walk into the unit, that this unit is currently studying X, and it has some bullet points under it about what Study X is.
Facilitating internal implementation	**Patient** We have on our Quality Improvement Board of Trustees reports from across the system, what's working, what isn't, innovative programs and how can we actually help facilitate this on a broader basis. Over the past 4 years, we've been making a very concerted effort of eliminating silos… and the keyword is standardizing, standardizing it throughout so it…s system wide, not just the best policy at this hospital… **Employee** And we should say that the Lean methodology that—we ran it here for over 6 years, and now it's really embedded in the culture. But we ran rapid improvement to that, that had over 575 staff members involved in this five‐year program of a Lean. And we also had patients in those events who really participated in the development of identification of problems and solutions.
Disseminating learning to others systems	**Patient** As part of my current advocacy work, I went to a Beryl patients' conference… to make a break‐out session presentation to about 100 people about our work. **Employee** Oh, that's all we do. I mean, it's trying to get that message out. We don't do it just to have a small group learn; we disseminate that. If one of our patient and family advisory councils comes up with a great idea, we implement it across our system at all our hospitals and ambulatory surgery sites, so, um, that's the whole message, and then, you know, we—like others across the country—you know, share at meetings, or an abstract and articles. Um, we share our learnings.

#### 3.1.1 Setting priorities

Patient leaders described working with system employees to set priorities for learning activities through brainstorming sessions, developing ideas in committee meetings, or participating in root cause analyses of serious safety events. Priorities also emerged out of research and QI processes that included patients “in the identification of what the issues are as well as in designing the countermeasures.” At a substantive level, patients consistently emphasized the importance of experiences with care as a focus and source for learning; in the words of one patient interviewee, “…at the forefront of our minds is always how do we improve the patient experience via quality and via safety?”

#### 3.1.2 Designing and conducting learning activities

“Engagement studios” at one LHS not only set priorities but also ensure input on pragmatic studies during the design phase, allowing researchers to “engage the people that you are actually going to be enrolling or studying in helping you develop that research.” Interviewees also described partnership on numerous interventions to improve health outcomes (eg, fewer blood clots) by facilitating hospitalized patients' ability to implement preventive measures and collecting data to measure comparative impact of such interventions across LHS units. Interviewees frequently described involvement in the conduct of research studies, particularly those funded by the Patient‐Centered Outcomes Research Institute (PCORI).

#### 3.1.3 Protecting patients' rights and interests

Engagement with respect to protecting the rights and interests of patients participating in learning focused both on informed consent to research and—as Exhibit 3 highlights—on strategies for increasing community‐wide awareness about LHSs' use of patient data for pragmatic studies.

#### 3.1.4 Facilitating internal implementation

In some LHSs, PFAC members share information about the same practice (eg, pain management, laboratory practices) and/or compare how well things are working by “looking at data [from]… different parts of the system and how well they're doing on this,” and then advocate for consistent implementation. Examples of success include strong patient partnership as part of one LHS's effort to “eliminate silos” and assure that High Reliability Organizations are “standardiz[ed] throughout so it's system wide,” and replicating a patient‐driven rare disease clinic model across additional clinical settings.

#### 3.1.5 Disseminating learning to others

Patients present successful learning activities in local, state, and national public forums. They also help train other institutions to replicate successful initiatives (see below for examples).

#### 3.1.6 Comprehensive engagement through patient‐led learning activities

Of particular note were descriptions of learning activities initiated and led by patients that came to include the elements of systematic learning paradigmatic of LHSs. In one system, for example, patients led the creation of a new clinic model for rare diseases. Their original motivation was to improve problematic experiences with care, but they successfully engaged with employee leaders to roll out measurement processes, internal implementation (eg, adaptation to other rare disease clinics; standardization of specific practices such as referrals), and external dissemination (eg, presenting nationally; training other LHSs to replicate the model). In another system, patient leaders developed a model for educating patients about postsurgical care and quality of life that evolved into guidance for clinical teams, an advisory council focusing on QI projects and process improvement, and funding for research studies that were later published.

### 3.2 Levels of patient engagement in governance vary

Interviewees' descriptions of practices and policy revealed significant variation in the extent to which patients engage in governance of learning. Below we describe the three engagement paradigms we discerned, Exhibit 4 provides a conceptual overview.

**EXHIBIT 4:**
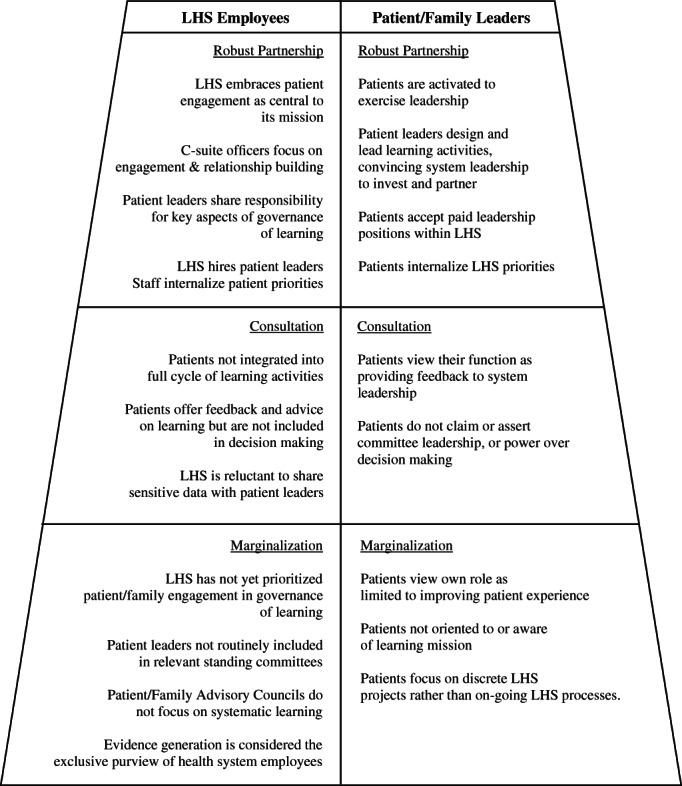
Levels of patient engagement in governance of learning

#### 3.2.1 Marginal involvement

In many LHSs, patient participation is limited to receiving information or to being the source of “data”—for example, via electronic health records or patient experience surveys. Our patient interviews evinced this paradigm primarily through limited participant awareness of the LHS's learning mission and the roles patients might play. Probes in our patient interview guide specific to learning were, in these cases, met with questions, confusion, or examples of involvement in discrete projects designed to improve patients' experience (eg, parking, facility renovations, welcome packets) rather than in governance of systematic learning. Some patients referred to not being “involved enough” to weigh in on PFAC agendas or project ideas.

Employee leaders were more often explicit about patients' marginal involvement. Some said that LHS engagement practices were not yet sufficiently sophisticated, that decision‐making about learning is the purview of “leadership… at the entity level” rather than of PFACs, or that priority‐setting is a function of “management and executive leadership.” Others stated that patients lack the requisite skills, knowledge, or background to participate in governance of learning activities; that LHS engagement should focus on making “complying with protocols… more user friendly;” or that LHSs consider it poor stewardship of volunteer time to make patient leaders “do the work” involved in governance functions.

#### 3.2.2 Consultative roles

Many interviewees described patients as playing consultative or partial rather than partnership roles with respect to governance of their LHS's learning mission. Patients noted that their PFACs are “on the fringes” of learning processes, give feedback regarding “serious safety events” but are not integrated into follow‐up learning activities, “…bring problems forward” so that other “appropriate people” can discuss them, or primarily provide feedback on recruitment materials and consent forms for research studies. Employee leaders emphasized the prevalence and persistence of consultative forms of engagement. As one put it, summarizing a perspective common in our interviews, “…what we do most of the time is we just develop something, and we get feedback from our customers, or our patients.”

#### 3.2.3 Robust partnership in governance

In some exemplary cases, patients described being woven firmly into critical governance structures such as system‐wide quality or safety committees, boards of directors, or leadership groups designated to oversee learning. As one patient put it, “… they fully trained me just like any manager so that I could work with… senior‐level members of the staff… as a partner.” Some patients played a direct role in facilitating meaningful engagement for others by advocating role expansion, creating effective recruitment and training initiatives, and learning to understand the priorities of institutional leaders and to speak in terms that institutional leaders can understand.

One employee leader, describing this end of the engagement spectrum, noted “… it's our mantra to include [patients] in all the work that we do… we involve them in continuous improvement…[we] actually get them into the project designing the work with us as opposed to just asking them intermittently what they think about it.” Others describe patients pushing for more aggressive quality and safety targets from within “a governance group…actually making decisions…[that] then go to our board for final approval,” and emphasize that patients are integral to identifying appropriate, patient‐centered process‐ and outcome‐level data. Institutions that robustly engage patients and families in governance give them at least some authority within a shared model of decision‐making.

### 3.3 Engagement is a perpetual work in progress

Both patient and employee interviewees emphasized the “never finished” nature of engagement work. Patients noted a “constant struggle” to determine where community voices are needed and to consistently remind employees that “there's never enough engagement with patients.” Others said discussions were actively underway about expanding patients' roles or including them on additional high‐level LHS committees. A shared governance model around learning “isn't something you attain,” said one patient; “it's something that you constantly work on and nurture and create a better relationship with…. It's kind of like cultural competency versus cultural humility.”

Employees described their LHSs as “on a journey of continuing to elevate” patient engagement in learning, using strategies such as culture change (see below), proving its value, or building on executive‐level interest in robust partnerships. Several noted how PCORI's funding requirements create useful impetus and proof of concept. One interviewee described how lack of an “enterprise‐wide governance system” around quality and safety directly impedes efforts to “includ[e]… the voice of the patient.” Employees in another system noted that though their LHS wants patients to be active in learning processes, in reality, the system is not “…transparent to the point in our quality or safety division that there's a lot of… customer input in terms of what they're trying to accomplish. But that's probably a great conversation starter.”

### 3.4 Supportive organizational culture is essential for shared governance of learning

Participants emphasized that organizational culture is essential to the “journey” of including patients in the governance of learning. One system's strategic plan explicitly embraced the goal of “more in‐depth engagement with patients as co‐investigators.” Other interviewees emphasized the importance of elements such as assurances of confidentiality, explicitly acknowledging the value of patients' perspective, approaching the process “one culture changer” at a time, and the key role of an empowered Office of Patient Experience. Participants also highlighted the importance of Federal agency guidance and requirements in promoting engagement with patients. Many noted the importance both of listening, respect, and creating a “comfortable environment” for dialogue between patients and employees and of “closing the loop” with advisors and ensuring their suggestions are taken seriously. In some LHSs, already‐engaged patients were integral to culture change; for example, they steadily pushed for more patient voice in governance structures, co‐led major events, chaired PFACs, or transitioned from unpaid advisory roles into paid staff positions.

Attributes that can enable or challenge a culture of robust shared governance are explored below. Exhibit 5 summarizes key findings, and supporting information included online provides illustrative quotes related to each attribute.

**EXHIBIT 5:** Challenges to and strategies for a patient‐engaged governance culture

Attribute	Challenges	Enabling strategies
Transparency	LHS reluctant to share sensitive data LHS concerned about bad press	Cultivating mutual trust between LHS and patient leaders with regard to sensitive data LHS views patient leaders as vital to learning from mistakes
Capacity building	No specific training to prepare for integration of patient perspectives No explicit, system‐wide commitment to a culture which acknowledges the value of patient participation in governance Engaging only patient members who already have research, quality improvement, or related skills	Training for both LHS employees and patient leaders System‐wide symposia and on‐going support for shared governance of learning Designated center for engaging patients and families
Committee structures	Patients restricted to PFACs PFACS have minimal influence Employees chair PFACs	Patients participate in quality, safety, adverse event, Board of Directors, and other powerful committees System‐wide PFAC Patients chair or co‐chair PFACs
Commitment from LHS leadership	LHS leaders not explicitly committed to engaging patients in systematic learning High leadership turn‐over Leaders not in direct contact with patients	LHS leaders prioritize deep engagement with patients in continuous improvement Patients and system leaders spend substantial time together Leaders assure patient input is taken seriously
Diversity and representation	LHS engages those most likely to volunteer or be nominated LHS counts business leaders on high‐level boards as “patients” because they use the health system for regular care	Engagement prioritizes patient and family leaders reflecting diversity of LHS's patient population Population‐specific PFACS established and invested in by institution and patient leaders Resources are dedicated to facilitating engagement (eg, stipends, travel, babysitting)
Development of system‐wide infrastructure for shared governance of learning	No system‐wide approach to shared governance of learning No C‐Suite level office of patient experience	LHS committed to systematic, strategic investment in shared governance of learning Designated office (eg, patient experience) or system‐wide initiative funded and tasked with systematically cultivating engagement

#### 3.4.1 Transparency

Patients noted that their roles could be limited by LHSs' reluctance to “admit publicly” that “there could be issues,” or by “worr[ies] about their [public relations].” Those same roles expand and become more meaningful when systems understand that transparency with patients is essential for “…continuing to improve our quality in the best way we can.” Employees voiced concern about showing the hospital's “dirty underwear” in front of patients and about the possibility that doing so might slow decision‐making. Others emphasized how hard it can be, stop thinking “everything needs to be perfect before we bring people in because we don't want people to see we don't have it all figured out.”

#### 3.4.2 Capacity building and training initiatives

Shared governance of learning requires both that patients examine issues systematically and that employees believe patients are capable of contributing to evidence‐driven practices. Given the complexity of the learning enterprise, sustained attention to capacity building and training for—and by—both patients and employees is essential. Toolkits, curricula, ongoing structured support for patients from committee leaders, and system‐wide symposia are prevalent strategies. One LHS has a Patient Experience Collaborative, co‐led by staff and patients, which works to develop best PFAC practices across the organization. Another “…striv[es] to be the one‐stop resource for embedding patient advisors in process improvement and research,” in part by implementing a targeted curriculum. Recruiting only participants already well‐versed in research or related fields presents challenges to the diversity of patient perspectives (see below) and designing curricula solely for patients “to be prepared to effectively give us their input” on quality issues leaves unaddressed the related need to train LHS employees “to hear the feedback from patients and families.”

#### 3.4.3 Committee structures

Participation in committees facilitated deep engagement when PFACs were empowered to make meaningful decisions, structure agendas, and connect with one another to share ideas and promote system‐wide improvement. Another effective strategy participants described is including patients as full members of standing committees such as those focused on quality, safety, or system‐wide oversight of learning. In some instances, patient members of these committees act as conduits for information, insights, and ideas by continuing simultaneously to sit on PFACs. In contrast, when patient participation is limited to PFACs and LHS employees drive PFAC agendas, consultative models tend to persist, limiting shared governance of learning.

#### 3.4.4 Leadership

Interviews highlighted the importance of support from high‐level LHS employees to a culture of shared governance over learning. Participation suffers when leaders rotate in and out of positions, under‐value engagement, or fail to spend time with individual patients and their committees. It thrives when patients regularly interact with “people who can make things happen,” when CEOs and other executives model respect for patients' contributions, when the LHS invests in a C‐suite office tasked with patient engagement and experience, and when the LHS hires patient leaders for high‐level positions.

#### 3.4.5 Diversity and representation

Both employee and patient participants emphasized the importance of skills, confidence, and diverse perspectives among patient leaders. Recruiting patients who are socioeconomically, racially, and ethnically diverse was identified as a challenge across the board, as was ensuring that diverse patients rather than “nonrepresentative” elite ones^25^ receive cherished high‐level appointments (eg, on LHS Boards of Directors). As one employee put it, describing having a single patient on the LHS's executive quality improvement committee, “I wouldn't say that [one patient] gives us five stars on having the patient perspective… you bring in a patient, and you have one patient's perspective.” Strategies for diversifying patient leadership included developing PFACs specifically for traditionally marginalized subgroups (eg, Latinx; youth; urban residents), using the Clinical and Translational Science Award's “special populations” mandate to build diverse community input and having patient leaders dedicated to diverse perspectives push the LHS to invest in solutions.

#### 3.4.6 Development of system‐wide infrastructure for shared governance of learning

Participants in several LHSs described the need for system‐wide infrastructure investment. One system built a dedicated center for patient engagement that generates “a pool of patient advisors” used system‐wide as “true partners with providers or administrators” for “process improvement, quality improvement, research, [and] participat[ion] on teams.” The center, which itself has substantial patient leadership, has trained and assigned more than 300 advisors to move, step by step, through various committee structures. Advisors first learn to represent a “patient eye view,” on lower level committees, then to turn their personal story into a “power story” that “serves as an example of how change can be made for … those that come after us” in PFAC settings, and then finally to “become representatives” who provide a “community eye view” on system‐wide committees (including the Board of Directors) and on issues such as what it means to be a high reliability organization. Another system is building out from an already‐comprehensive PFAC system to “make an online community… potentially having thousands of advisors…” who can weigh in remotely on salient issues, and has hired patient leaders to run and staff a C‐suite‐level Chief Experience Officer office with the remit to “… make sure that they always keep the patient family voice in every aspect of the hospital.”

## 4 DISCUSSION

In order for LHSs to realize their potential to improve efficiency, cost, and quality in health care, it is essential to determine “… how patients can drive transformation toward continuous learning.”^11^ As LHSs across the country continue to move engagement “from ethical frameworks to practical implementation,”^10^ it is clear that if patients are to have substantial impact, they must be engaged in governance rather than serving only as data sources or sounding boards. Findings from our study provide both an empirically grounded overview of where efforts to engage patients in governance of learning stand and concrete examples of what facilitates or impedes their playing such a role.

Our data show that, despite widespread support for patient engagement in governance of learning, such engagement exists along a continuum that ranges from minimalist through consultative to robust partnership (see Exhibit 4). While our interviews emphasized institutional structures and norms as key factors inhibiting or promoting engagement, they also highlighted (as does seminal work regarding co‐production of healthcare more generally^26^) the active role patients can play in moving systems toward shared governance.

Existing scholarship suggests that, once activated, patients can grow in their commitment to a “shared learning journey” and that their social identities can evolve from consultant to full partner. ^15^, ^27^ Our study highlights elements of organizational culture—including transparency, capacity‐building, leadership, attention to diversity and representation, and infrastructure investments—that promote travel along the pathway to shared governance of learning processes. Our data also indicate that, even for those systems that have made the most progress, achieving the ideal of robust patient engagement in governance of learning remains a work in process.

Many of the same issues vexing robust patient engagement in health care generally—including representation, diversity, expertise, legitimacy, and the challenges of overcoming long‐standing norms and culture ^20^, ^21^, ^28^—consistently arise for LHSs. Their size and complexity also present specific challenges, as does their focus on continuous learning.

Nonetheless, useful exemplars emerged from our interviews. Leaders in systems that partner with patients say that the insight, ideas, and capacity patients bring to learning highlight quality issues that require attention, motivate needed changes in workflow, and allow for the LHS to genuinely focus on “that journey for… patient‐centered care.” Patients say it is deeply empowering to “drive healthcare change and outcome from a patient… perspective.” Working together, employees and patients can successfully learn how to move LHSs closer to their goal of high‐quality, efficient, and effective care.

## CONFLICT OF INTEREST

Dr Steve Joffe received research funding from Pfizer for a project unrelated to the work reported in the research article submitted here. That grant went through Penn and ended May 2020. No other authors have conflicts of interest to declare.

## Supporting information


**Appendix S1.** Supporting Information.
