# Supplementary material for: Patients' roles in governance of learning: Results from a qualitative study of 16 learning healthcare systems
Source: Learn Health Syst. 2021 May 25;6(1):e10269. doi: 10.1002/lrh2.10269 (PMC8753297; doi:10.1002/lrh2.10269)
Supplement: Supplementary file 1 — Appendix S1. Supporting Information. [file LRH2-6-e10269-s001.docx]

**Supporting Information**

**Challenges to and Strategies for a Patient-Engaged Governance Culture: Illustrative Quotes**

| Attribute | Challenges | Enabling Strategies/Responses |
| --- | --- | --- |
| Transparency | So, I think the biggest challenge is talking through people’s fears of doing that. Like if I put someone on a grievance committee, they’re gonna hear all the not so great stuff that happens and talking through that with someone. Like what are the pluses to doing this, what are the negatives? What makes us nervous and can we start to be much more candid with the people that we ask to be in that space with us? | And by allowing us to see that and be open and willing to see where those faults are, it almost makes me appreciate and love the institution more, and the work that they're doing because they're not like, "Oh yeah, we're perfect," and then underneath the sea, there is a lot of really awful stuff going on that they just manage to hide from people. |
| Capacity Building | And she tapped me on the shoulder and said… we’re looking for a [patient] to sit in on a really high-powered steering committee that reviewed research that the [organization] is doing and really muckety muck with some guys for 8 to 10 hours with the researchers. And I said, that sounds fascinating… so I ended up on that steering committee…. I didn’t have the foggiest idea what maybe the research was, it was just so deep. | I think [patients] can have a huge impact… [But]… how much impact they have really depends on the ability of the leader and the clinicians in the area in which they're working to effectively deploy that feedback. That's why we try to prepare families to be able to give their feedback in ways that will be more likely to be heard, and we train the clinician in that particular area to hear the feedback from patients and families…. |
| Committee Structures | In terms of the overall governance structure, I don’t know of any of our clinical programs that has a – a sitting and permanent – semi-permanent patient member. Probably that’s something that’s in the future as well, but, um, I don’t know of it yet. | …topics are brought up through the, um, the regular PFACs through the system-wide PFACs. They’re discussed at that system-wide PFACQS, and then, we, we kind of go execute….. [The VP for Quality and Safety and the Safety Consultant] are strong supporters of all of the system PFACQs. |
| Commitment from LHS Leadership | Um, and so, one of the barriers are, is that appreciated at the most senior level? | The support of senior leadership and the respect for patient advisors is one of the primary ingredients for successful patient input. Having that respect, having senior leadership incorporate it and show that by role modelling it. |
| Diversity of patient leadership | But I know, for example, that our FAB is made up of a lot of white women. We are moms, and most of us are in careers, either stay at home or in career that we can take time off. And there is a huge population at [LHS] of color, of different backgrounds, language, accessibility, different socioeconomical backgrounds, and I know that there are just challenges to getting multiple viewpoints involved. | Well, I know they have developed a Patient Advisory Board using teenagers to look at some issues involving older adolescents to get their feel on things. |
| System-wide Infrastructure Investment | [T]hey’re not operating from a plan, from a vision, they’re operating ad hoc, whatever comes up is kind of what they deal with. They hear a new program’s starting, they’ll say, oh, can somebody call them and have them come in and talk next month. So, I think their structure is just a little Loosey Goosey… it’s not created as a tool that leverages the strength of [patients/families] who know the hospital in association with the contact that they have with management.... | It’s certainly a priority for us to have patients and community members in every aspect of our work, and we have developed standards and expectations around that engagement for them so that they have to be paid, there has to be a system in place to balance power, and give them voice, and to make sure that they have an opportunity to provide input, and also evaluate the process. |
